# COVID-Induced Hyperthyroidism in a 30-Year-Old Female: A Case Study

**DOI:** 10.7759/cureus.40851

**Published:** 2023-06-23

**Authors:** Benjamin Ilyaev, Sabina N Muminiy, Emmanuella Borukh, Emmanuel Izrailov, Yakubmiyer Musheyev, Stella Ilyayeva

**Affiliations:** 1 Medicine, Hofstra University, Hempstead, USA; 2 Medicine, St. Francis College, New York City, USA; 3 Medicine, Yeshiva University, New York City, USA; 4 Medicine, Touro College, New York City, USA; 5 Medicine, New York Institute of Technology College of Osteopathic Medicine (NYITCOM), Old Westbury, USA; 6 Endocrinology and Diabetes, Atlantic Endocrinology and Diabetes Center, New York City, USA

**Keywords:** sonogram, thyrotoxicosis, thyroiditis, covid-19, covid induced hyperthyroidism, hyperthyroidism, thyroid disorder, clinical endocrinology

## Abstract

Thyroiditis should be in the differential for hyperthyroidism when thyroid-stimulating hormone (TSH) is suppressed and T3/T4 levels are elevated. Suspicion of hyperthyroidism is further increased when the patient can exhibit symptoms such as weight loss, anxiety, feeling feverish, tremors, shaking, and sweating. Hyperthyroidism is generally classified as being overt or subclinical. In the following case, the patient had overt hyperthyroidism which is considered more severe than subclinical hyperthyroidism. Coronavirus disease is not typically associated with thyroiditis; however, in this case, the patient's disorder was accidentally found on her blood results which were originally taken due to her coronavirus disease 2019 (COVID-19) diagnosis.

In this case study, we present a 30-year-old female patient, with suspicions of COVID-19-induced hyperthyroidism found incidentally on her blood panel. An original diagnosis of thyroiditis was made prior to the visualization of increased release of thyroid hormone. A sonogram was done, and a follow-up blood panel was ordered, confirming the patient’s diagnosis of hyperthyroidism post COVID-19 recovery.

## Introduction

Coronavirus disease 2019 (COVID-19) is a highly contagious infectious disease caused by severe acute respiratory syndrome coronavirus 2 (SARS-CoV-2) [1]. Symptoms of COVID-19 are multiple and highly heterogeneous in their distribution. These include fever, cough, fatigue, anorexia, shortness of breath, sputum production, and myalgia [2]. Nucleic acid detection techniques, like RT-PCR, are considered an effective method for confirming the diagnosis in clinical cases of COVID-19 [3].

One of the first systems affected by COVID-19 is the respiratory system. However, it has become increasingly clear that its effects extend beyond that of the respiratory system [4]. There is documentation of thyroid and pituitary disruption in patients with COVID-19, which has resulted in significant interest in its impact on the endocrine system [4]. More specifically, COVID-19 has been documented to decrease thyroid-stimulating hormone (TSH) levels and induce thyrotoxicosis, destructive thyroiditis, and de novo Graves’ disease [5].

Hyperthyroidism, otherwise known as thyrotoxicosis, occurs when excess thyroid hormone is released passively or due to an overactive thyroid [6]. Examples of such thyroid hormones are triiodothyronine (T3) and thyroxine (T4), both of which function to regulate metabolism. Generally speaking, hyperthyroidism is classified as being either overt or subclinical. In cases of overt hyperthyroidism, thyrotropin (TSH) levels are suppressed while T3 and free T4 levels are high. In subclinical hyperthyroidism, TSH levels are still low or suppressed; however, T3 and free T4 levels are within the standard range [6]. The overall prevalence of hyperthyroidism in the United States is 1.2%, with 0.5% of cases consisting of overt hyperthyroidism and 0.7% of cases consisting of subclinical hyperthyroidism [6]. Hyperthyroidism also has a range of symptoms including but not limited to weight loss (due to increased metabolism), increased appetite, exophthalmos (bulging eyes), periorbital edema, palpitations, atrial fibrillation (AF), tachycardia, insomnia, and anxiety [7]. Common causes of hyperthyroidism are Graves' disease, toxic multinodular goiter, toxic adenoma, and painless viral thyroiditis. 

## Case presentation

A 30-year-old female presented to her endocrinologist with complaints of neck pain, difficulty swallowing, tremors, shaking, sweating, weight loss, anxiety, and fever. These symptoms developed after contracting a COVID-19 infection, confirmed by a PCR test and resolved within two weeks. A positive COVID test warranted a complete blood panel, and the results revealed suppressed TSH and elevated free T4, warranting a comprehensive endocrine consultation in order to rule out thyroid disease.

She had a family history of diabetes and no known family history of thyroid disease, while her social history indicated nothing of significance as for diet, exercise, and stress levels. Her past medical history showed no indication of any thyroid disorder. At the time of her endocrine consultation, 600mg Ibuprofen tablets were being taken with meals, three times a day, in order to aid in pain mitigation. 

Ten days after the initial suspicion of hyperthyroidism, a sonogram was done. The test revealed that the patient's thyroid gland was normal in size; however, both lobes of the thyroid gland had diffusely heterogeneous echotexture, and increased vascularity was noted in both lobes of the thyroid gland which caused suspicions of COVID-19-induced viral thyroiditis to be discussed. The patient's blood reports indicated an abnormal white blood cell count, suppressed TSH, and elevated free T4, erythrocyte sedimentation rate (ESR), and C-reactive protein (CRP). It also showed results below the normal range for thyroid peroxidase AB and thyroglobulin AB.

The differential diagnosis for a patient presenting with symptoms of thyroiditis, suppressed TSH, elevated free T4, ESR, CRP, and white blood cell count after experiencing viral COVID-19 infection is consistent with COVID-induced viral hyperthyroidism. 

**Figure 1:**
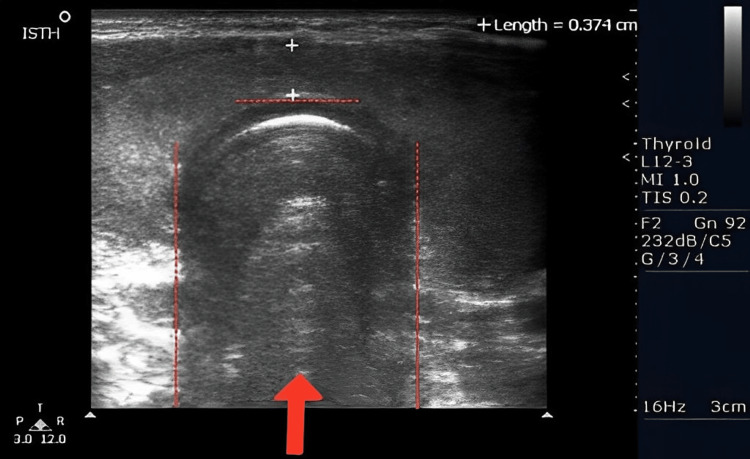
This figure depicts the whole isthmus. The red arrow and the region inside the dotted lines are the isthmus noted in the sonogram scan.

**Figure 2:**
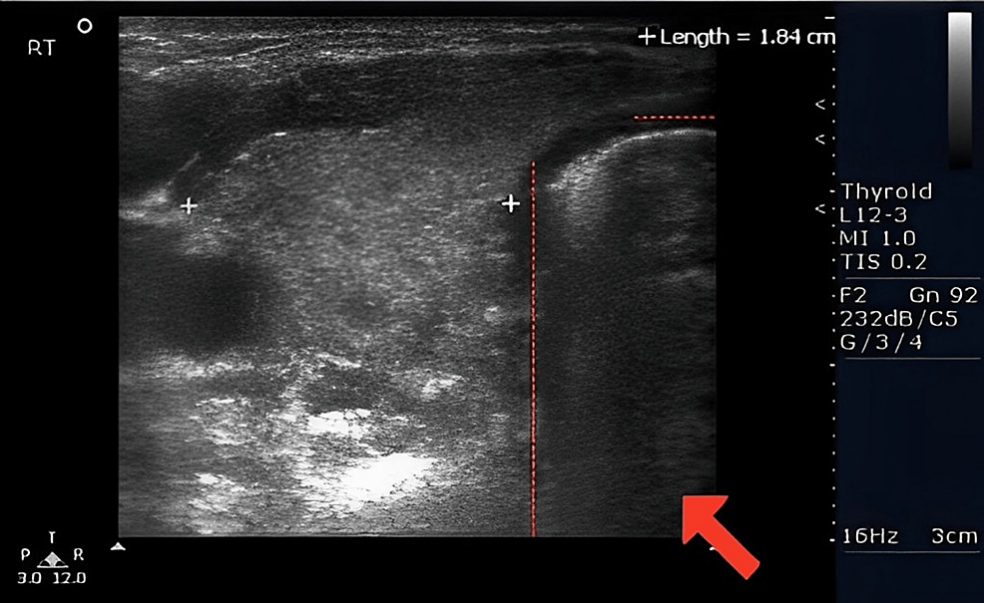
The right side of the isthmus is depicted. The dotted lines depict the perimeter of the isthmus and the red arrow points toward the location of the isthmus.

**Figure 3:**
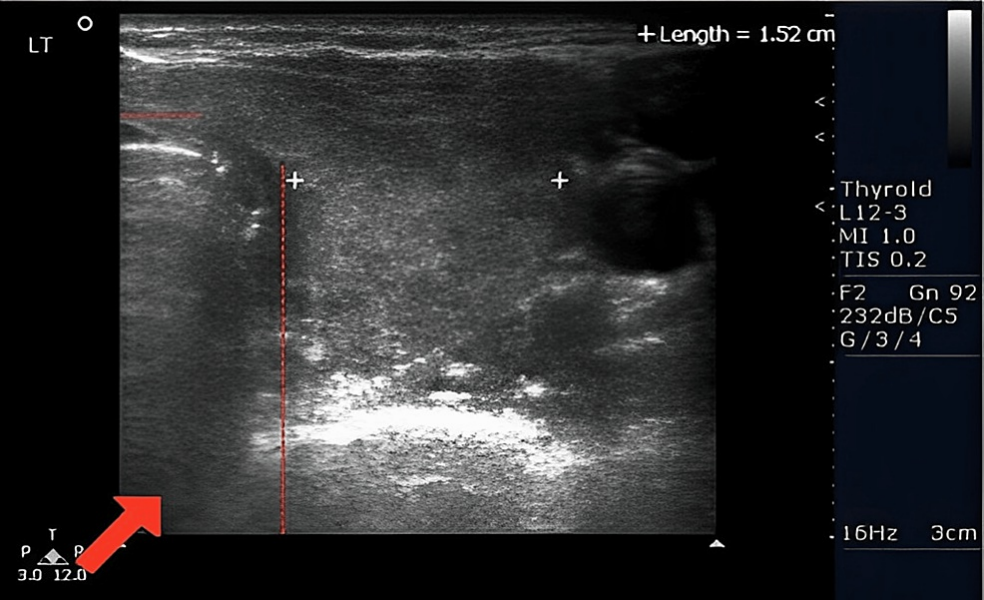
The left side of the isthmus is depicted. The dotted lines depict the perimeter of the isthmus and the red arrow points toward the location of the isthmus

The patient underwent a sonogram of the thyroid gland 11 days after initial presentation of thyroiditis suspicions (Figure 1). Findings showed that the thyroid gland was normal in size with the right heterogeneous lobe measuring 3.7 x 1.3 x 1.8 cm (Figure 2) and a left heterogenous lobe measuring 3.9 x 1.6 x 1.5 cm (Figure 3), neither showing nodules or cysts. The isthmus measured 0.4 cm. The sonogram technician noted echogenicity was normal and increased vascularity in both lobes, and the thyroid appears to have a diffusely heterogeneous texture. These findings, in addition to the abnormalities indicated on the patient's complete blood panel, indicated hyperthyroidism. 

The patient originally tested positive for COVID-19, warranting a full blood panel, and revealed endocrinological abnormalities. Specifically, suppressed TSH levels and elevated T4 levels indicated the need for further evaluation by the patient's endocrinologist. A sonogram conducted 11 days later during the follow-up further suggested abnormalities in the lobes of the thyroid gland. In addition, a second blood panel, completed to confirm the endocrinologist's suspicions, illustrated continued abnormalities in TSH and T4 levels, as well as elevated white blood cells, ESR, and CRP. The patient was diagnosed with COVID-induced hyperthyroidism.

The standard treatment for hyperthyroidism is antithyroid medications, radioactive iodine ablation, or surgical thyroidectomy [5]. After the initial appointment confirming endocrine intervention was necessary, a follow-up was scheduled to perform a thyroid sonogram eleven days later. At the follow-up, the sonogram showed a diffusely heterogeneous thyroid gland with increased vascularity and the endocrinologist could not rule out thyroiditis. The patient experienced symptoms consistent with a thyroiditis diagnosis and could not swallow due to severe inflammation of the thyroid. This led to a prednisone prescription as an initial course of treatment in order to decrease the swelling. Another blood panel was done and the results, discussed over the phone with the patient, confirmed COVID-induced hyperthyroidism. The endocrinologist and medical team continued to check up on the patient over the coming weeks to monitor symptoms.

## Discussion

The term hyperthyroidism refers to any condition in which an overactive thyroid gland produces excess levels of T3 and T4 thyroid hormones that play a significant role in regulating metabolism [6]. As a result, patients may experience all or some of the following symptoms of anxiety, excessive sweating, tremors, weight loss, exophthalmos, tachycardia, insomnia, increased appetite, periorbital edema, palpitations, and atrial fibrillation [7]. Upon visualization of symptoms, blood work was done. Elevated levels of ESR and CRP, in addition to the other noted symptoms, were indicative of thyroiditis, and a sonogram was suggested to be done. These results would not confirm the diagnosis until a thyroid scan with iodine uptake is done, in order to rule out high uptake. 

Recent research has shown that the outbreak of SARS-CoV-2 might have a significant impact on the functions of the endocrine system [4]. COVID-19 has been linked to being responsible for the initiation of a cytokine storm which contributes to some of the more severe pathologies associated with it such as acute respiratory distress syndrome, multi-organ failure, and in particular autoimmune diseases [5]. In particular, these effects of COVID-19 were observed in disruptive changes that occurred in the thyroid gland [5]. It has been shown to decrease TSH levels and cause de novo Graves' disease, thyrotoxicosis, and destructive thyroiditis. Other literature additionally suggests that on a cellular level, the SARS-CoV-2 spike protein gains access to the host cell through the process of binding to the ACE-2 receptor [1]. In the human body, the endocrine system contains an abundance of ACE 2 receptors becoming the target of SARS-CoV-2 [5]. In the case presented, the patient started getting symptoms of thyroid dysfunction after being tested positive for COVID-19. The symptoms of anxiety, weight loss, pain in the throat, difficulty swallowing, tremors, and fever suggested potential thyroid dysfunction. The blood panel revealed elevated levels of ESR and CRP indicative of inflammation via viral infection; however, the results also showed abnormal levels of TSH and T4 hormones consistent with thyroiditis, suggesting hyperthyroidism. 

Upon confirming the diagnosis, physicians focus on identifying the underlying cause of hyperthyroidism, which may include Graves' disease, toxic multinodular goiter (Plummer disease), thyroid adenomas (TAs), or thyroiditis [6]. Graves' disease is the most common cause of hyperthyroidism and is an autoimmune disorder in which TSH receptors are activated by thyroid-stimulating antibodies thereby inducing thyroid hormone synthesis [6]. Plummer disease is the second most common cause of hyperthyroidism and characterized by hormonally hyperactive (autonomic) multinodular goiter [8]. It can manifest as low TSH levels and subclinical hyperthyroidism but lead to thyrotoxicosis if left untreated. Unlike Plummer disease, TAs are generally benign lesions that can be both active and inactive [8]. Most of the time, patients present asymptomatic and clinically euthyroid; only 1% of patients with adenomas are hyperthyroid and may experience symptoms corresponding to hyperthyroidism [9]. Finally, thyroiditis is characterized by the inflammation of the thyroid gland and can cause both transient and chronic hypothyroidism (Hashimoto's disease) or hyperthyroidism. Therefore, health care providers consider checking thyroid peroxidase antibodies (TPO) early on to rule out the possibility of thyroiditis-caused Hashimoto's disease. 

Furthermore, determining the thyroid gland's morphology by conducting a sonogram helps determine the presence of any nodules or cysts as well as the overall size of the thyroid gland [10]. However, despite the size of the gland and the absence of any nodules, it is imperative to note the vascularity of the gland. In our case, despite the normal size of the thyroid gland and the absence of any nodules and cysts, increased vascularity of both lobes was noted. The results of the bloodwork and thyroid sonogram were discussed with the whole medical team; it was agreed that the patient developed COVID-19 virus-induced hyperthyroidism. 

The therapy of hyperthyroidism is contingent upon the disease's source and severity, as well as the patient's age, comorbidities, and therapeutic preferences. The purpose of treatment is to correct the hypermetabolic state with minimal side effects and minimal hypothyroidism incidence. The most common treatments for persistent hyperthyroidism are antithyroid medications, thyroid surgery, and radioactive iodine [7]. The cause of hyperthyroidism could be identified by a radioactive iodine uptake test and a thyroid scan. By observing the percentage of the radioactive iodine taken up by the thyroid gland (also known as the uptake), doctors can determine the cause of hyperthyroidism [6]. In the discussed case, an iodine uptake was not done, but it is important to note if done, it would have shown decreased uptake. 

Since, in this case, the patient was diagnosed with thyroiditis accompanied by severe swelling and aggressive pain in the neck, she was treated with pain relief and low-dose corticosteroid medications, specifically prednisone, to ameliorate the symptoms and bring the levels of T4 down and normalize TSH. Prednisone was prescribed by the endocrinologist to decrease the inflammation of the thyroid gland due to the patient's pain in the neck [11]. 

## Conclusions

This study outlines the symptoms, diagnoses, and treatment approaches for a patient with hyperthyroidism. In particular, it focuses on COVID-19-induced thyroiditis of a female patient with no family history of thyroid dysfunction or symptoms of hyperthyroidism before contracting a viral infection. This case has shown the importance of screening patients for endocrine disorders after COVID-19 infection, especially if patients present with potential endocrine disorder symptoms. The most efficient diagnosis methods are laboratory tests focusing on thyroid hormones such as T3 and T4 in addition to the TSH levels. Also, physicians should consider sonogram imaging to determine the cause of the problem, as it may be crucial in identifying the cause of hyperthyroidism. Thus, a physician should be allowed to provide proper treatment to return thyroid function and hormone levels to normal. 

## References

[1] Cascella M, Rajnik M, Aleem A, Dulebohn SC, Di Napoli R (2023). Features, evaluation, and treatment of coronavirus (COVID-19). StatPearls.

[2] Aimrane A, Laaradia MA, Sereno D (2022). Insight into COVID-19's epidemiology, pathology, and treatment. Heliyon.

[3] Dhama K, Khan S, Tiwari R (2020). Coronavirus disease 2019-COVID-19. Clin Microbiol Rev.

[4] Clarke SA, Abbara A, Dhillo WS (2022). Impact of COVID-19 on the endocrine system: a mini-review. Endocrinology.

[5] Mehta A, Andrew Awuah W, Yarlagadda R (2022). Investigating thyroid dysfunction in the context of COVID-19 infection. Ann Med Surg (Lond).

[6] Reddy V, Taha W, Kundumadam S, Khan M (2017). Atrial fibrillation and hyperthyroidism: a literature review. Indian Heart J.

[7] Kravets I (2016). Hyperthyroidism: diagnosis and treatment. Am Fam Physician.

[8] Khalid N, Can AS (2023). Plummer disease. StatPearls [Internet].

[9] Mulita F, Anjum F (2023). Thyroid adenoma. StatPearls [Internet].

[10] Fariduddin MM, Singh G (2023). Thyroiditis. StatPearls [Internet].

[11] Koirala KP, Sharma V (2015). Treatment of acute painful thyroiditis with low dose prednisolone: a study on patients from Western Nepal. J Clin Diagn Res.

